# RE: Parasacral transcutaneous electrical nerve stimulation in children with overactive bladder: comparison between sessions administered two and three times weekly

**DOI:** 10.1590/S1677-5538.IBJU.2021.0341

**Published:** 2021-05-20

**Authors:** Johnnatas Mikael Lopes, Eldys Myler Santos Marinho, Rodolpho Nunes

**Affiliations:** 1 Universidade Federal do Vale do São Francisco Pós-graduação em Educação Física Paulo AfonsoBA Brasil Pós-graduação em Educação Física, Universidade Federal do Vale do São Francisco - UNIVASF, Paulo Afonso, BA, Brasil.

*To the editor,*

The management of overactive bladder in children is a multidisciplinary approach that requires complementary interventions. The study by Veiga et al. (1) evidences the possible effectiveness of transcutaneous electrical nerve stimulation (TENS) in the parasacral region with two (2W) and three (3W) times per week of intervention. This research was published in volume 47, n. 4, of this journal.

The results of Veiga et al. (1) need to be further debated in order to make better inferences. Methodologically, the absence of a placebo control group makes it impossible to state that the therapeutic effects achieved are outside the spectrum of the pathological natural history itself or by the action of urotherapy administered to both groups. (2) Answering these questions would be left only in the field of conjecture or in the confidence in other experiments with the method employed.

However, using the information provided by the authors in their results, it is possible to expand the discussion. We will analyze the results of Table-2 of the study by Veiga et al. (1), where three variables of the voiding diary were examined (urinary frequency, mean volume of urine eliminated and maximum volume of urine eliminated) and analyzed by t test and Wilcoxon test, as reported in methods. This test applies to intergroup and intragroup analysis, respectively.

It is possible to estimate the 95% confidence intervals (95% CI) of the groups using the means and standard deviations provided (Table-1). We noticed that the 2W group with TENS is statistically different from the 3W group in terms of all the variables of the voiding diary in the pre-intervention moment. Even so, the 2W group of TENS showed a statistically significant and clinically moderate (d=0.40) reduction in urinary frequency as well as an increase in the average urinary volume (d=-0.40) and in the maximum volume of urine (d=-0.33) after the intervention. These probabilistic and clinical impact modifications were not observed in the 3W group.

**Table 1:** Comparison of the variables of the voiding diary between the groups of two and three times a week of TENS.

	Two times			Three Times		
	Before	After	Cohen’ d	Before	After	Cohen’ d
Urinary frequency	10.5±3.7 (IC95%:8.68-12.18)	6.7±1 (IC95%:6.21-7.19)	0.40	7.2±0.7 (IC95%:6.88-7.52)	7.1±0.6 (IC95%:6.83-7.37)	0.03
Mean volume of urine voided (mL)	97.4±18.2 (IC95%:95.18-99.62)	132.5±23.7 (IC95%:120.89-144.11)	-0.41	117±13.4 (IC95%:110.81-123.19)	126.7±16.9 (IC95%:118.89-134.50)	-0.16
Maximum volume of urine voided (mL)	186.2±34.7 (IC95%:169.19-203.20)	241±47.7 (IC95%:217.63-264.37)	-0.33	248.6±43 (IC95%:228.74-268.46)	233.6±21.6 (IC95%:223.62-243.58)	0.11

*Adapted from Veiga et al.(**1**)*

The application of the t test for intergroup analysis and Wilcoxon for intragroup analysis would not reveal these findings because they are not the best analysis strategy. (3) The approaches with post hoc analysis would produce control of the biased analyzes multiple. In addition, it is important to have the aid of measures of clinical effect such as Cohen's d or its similar (4).

From this analysis, the questions arise: why does TENS 2W have better effects than 3W? Does TENS have a negative effect with 3W? Probably the best answer is that there is a main effect or interaction with urotherapy common to the groups and that perhaps it was not administered equally to the groups. It is also possible that the older age in the 3W group reveals influence.

Therefore, with the findings of the present study, we cannot conclude the effect of TENS in the voiding diary variables in children with overactive bladder due to the lack of a control group, just as we cannot say that 2W is better than therapy 3W due to the effects of other therapies/variables.

The Authors
